# Applying Fully Convolutional Architectures for Semantic Segmentation of a Single Tree Species in Urban Environment on High Resolution UAV Optical Imagery

**DOI:** 10.3390/s20020563

**Published:** 2020-01-20

**Authors:** Daliana Lobo Torres, Raul Queiroz Feitosa, Patrick Nigri Happ, Laura Elena Cué La Rosa, José Marcato Junior, José Martins, Patrik Olã Bressan, Wesley Nunes Gonçalves, Veraldo Liesenberg

**Affiliations:** 1 Department of Electrical Engineering, Pontifical Catholic University of Rio de Janeiro, Rio de Janeiro 22451-900, Brazilraul@ele.puc-rio.br (R.Q.F.); patrick@ele.puc-rio.br (P.N.H.);; 2 Faculty of Engineering, Architecture and Urbanism and Geography, Federal University of Mato Grosso do Sul, Campo Grande 79070-900, Brazil; jose.marcato@ufms.br (J.M.J.); jose.a@ufms.br (J.M.); wesley.goncalves@ufms.br (W.N.G.); 3 Federal Institute of Mato Grosso do Sul, Jardim 79240-000, Brazil; patrik.bressan@ifms.edu.br; 4 Faculty of Computer Science, Federal University of Mato Grosso do Sul, Campo Grande 79070-900, Brazil; 5 Department of Forest Engineering, Santa Catarina State University, Lages 88520-000, Brazil

**Keywords:** deep learning, fully convolution neural networks, semantic segmentation, unmanned aerial vehicle (UAV)

## Abstract

This study proposes and evaluates five deep fully convolutional networks (FCNs) for the semantic segmentation of a single tree species: SegNet, U-Net, FC-DenseNet, and two DeepLabv3+ variants. The performance of the FCN designs is evaluated experimentally in terms of classification accuracy and computational load. We also verify the benefits of fully connected conditional random fields (CRFs) as a post-processing step to improve the segmentation maps. The analysis is conducted on a set of images captured by an RGB camera aboard a UAV flying over an urban area. The dataset also contains a mask that indicates the occurrence of an endangered species called *Dipteryx alata* Vogel, also known as cumbaru, taken as the species to be identified. The experimental analysis shows the effectiveness of each design and reports average overall accuracy ranging from 88.9% to 96.7%, an F1-score between 87.0% and 96.1%, and IoU from 77.1% to 92.5%. We also realize that CRF consistently improves the performance, but at a high computational cost.

## 1. Introduction

Forest monitoring provides essential information to support public policies related to protection, control, climate change mitigation, and sustainable development. Therefore, the continuous monitoring of forest trends through remote sensing enables a cost efficient measurement of vegetated ecosystems. In this context, satellite observations constitute a suitable platform to cover large areas at regular periodicity [1].

In remote sensing, object detection is a common and challenging problem aiming to locate instances of a given object class in a specific image [2]. In the forest monitoring context, single tree detection is an essential task for many applications, including resource inventories, wildlife habitat mapping, biodiversity assessment, and hazard and stress management [3]. Over the years, researchers have worked in this field, mapping single tree species based on different satellite imagery and achieving moderate results [4,5,6,7,8]. In the last decade, new approaches emerged to take advantage of the characteristics of active sensors, especially light detection and ranging (LiDAR) systems, which became a trend for tree crown detection [9]. More recently, the authors in [10] concluded that combining LiDAR data with optical imagery generally leads to better classification accuracy. Although this conclusion might be generalized, the authors focused on classifying tree species in urban environments.

In fact, urban forests are a particular case of forests with singular attributes and peculiarities. Urban forests are commonly defined as woody vegetation located in an urban area and usually limited to single and/or groups of trees distributed in parking places, gardens, small parks, and along roads in the city. They may be associated with flower beds or be in contrast to grass and herbaceous shrubs [11]. Thus, the heterogeneity of urban environments makes the accurate classification of tree species more challenging than in natural forests. Firstly, a high spatial resolution image is required in order to differentiate them as individual objects. Secondly, with the progress of urbanization, urban trees are heavily influenced in their environment by urban patterns like streets, communities, and factories [12].

More recently, unmanned aerial vehicles (UAVs) can provide appropriate temporal and spatial resolution images to produce suitable datasets for mapping forested areas on the individual tree level [13]. This may allow a better detection of single trees in urban scenarios.

The flexibility, versatility, and low cost, as well as the recent advances in high spatial resolution cameras [13] have spread the use of UAVs in a wide range of applications, like precision agriculture [14,15] and ecological, environmental, and conservation monitoring [16,17,18]. Following this trend, Feng and Li [19] proposed a method for mapping tree species in urban areas based on histograms and thresholding using UAV observations. Similarly, Baena et al. [20] used object based image analysis on high spatial resolution UAV images to identify and quantify tree species across different landscapes. On the other hand, computer vision has evolved substantially in the last decade, mainly due to the introduction of deep learning methods. In this context, convolutional neural networks (CNNs) have become the most common approach for different image analysis tasks such as automatic classification, object detection, and semantic segmentation [3,5,6,21,22,23,24,25]. Recently, CNNs have been widely applied for remote sensing problems achieving the state-of-the-art in many applications [26]. Some deep learning based approaches for tree species detection have been proposed in recent years. Li et al. [27] presented a deep learning based framework for oil palm tree detection and counting, using high spatial resolution satellite images. Weinstein et al. [28] used RGB images from an airborne observation platform along with airborne LiDAR data to detect tree crowns through a deep learning network.

Considering UAV platforms, Natesan et al. [29] proposed a deep learning framework for tree species classification. In this approach, images of pre-delineated tree crowns were the inputs to a CNN to classify the delineated trees to one out of three classes: red pine, white pine, and non-pine. Similarly, Masanori et al. [30] used UAVs to acquire RGB images of individual tree crowns and carried out a multiresolution segmentation algorithm [31] to classify seven different types of trees. Overall accuracy up to 89% was reported in this study. In [21], Santos et al. proposed different deep learning methods for detecting law protected tree species using high resolution RGB imagery. These methods delivered a bounding box that enclosed each object instance, but did not delineate the shape or contour of the target. In contrast, semantic segmentation is the task of assigning a class label to each pixel in the image [32]. Thus, semantic segmentation has the potential to capture object form and size more accurately than object detection, which may be essential in many applications.

The first idea for deep semantic segmentation methods was to build a patch based CNN. This approach consists of splitting the image into patches and classifying their central pixel using a traditional CNN. A critical drawback of this method is the redundant operations, specifically in overlapping patches, associated with its high computational cost. To overcome these difficulties, fully convolutional neural networks (FCNs) were first proposed in [33]. The network uses convolutional and pooling layers to build an end-to-end network able to manage different spatial resolutions and predict class labels for all pixels, exploiting context and location information of the objects in the scene. Later on, with U-Net [34], a technique to improve the spatial accuracy of the segmentation outcome was proposed. Typically, in this approach, the input image is first processed by an encoder path consisting of convolutional and pooling layers that reduces the spatial resolution. It is then followed by a decoder path that recovers the original spatial image resolution by using upsampling layers followed by convolutional layers (“up-convolution”). In addition, the network uses the so-called skip connections appending the output of the corresponding layers in the encoder path to the inputs of the decoder path. The SegNet architecture [24], as the U-Net, employs the same principle of the encoder and decoder paths. However, instead of using skip connections, the decoder makes use of the pooling indices computed in the pooling operation of the corresponding encoder layers to upsample the result up to the original image resolution. Recently, Mask R-CNN [35] combined both detection and segmentation in an end-to-end fashion. Beyond predicting the class and the object bounding box, as required by the detection task, the network also outputs the binary object mask. Mask R-CNN was designed for instance segmentation. Strictly speaking, this application is different from the one addressed by the present study. In fact, the Mask R-CNN also uses an FCN which, however, only segments the region within the predicted bounding boxes.

Some authors proposed the use of a conditional random fields (CRF) based post-processing to further improve the spatial and semantic accuracy of the FCN outcome (e.g., [25,36]). Notwithstanding the reported improvements brought about by CRF, these methods have a significant drawback: FCN and CRF need to be trained separately so that such methods constitute no end-to-end solution. In the last few years, real end-to-end FCN architectures for semantic segmentation were published, which reportedly performed at least as good as prior solutions that included CRF post-processing (e.g., [37,38]). This was achieved due to innovative techniques to capture multi-scale context within the FCN, such as global-to-local contexts aggregation as in ScasNet [37] and atrous spatial pyramid pooling in DeepLabv3+ [38].

In recent years, a few studies have already evaluated the potential of the FCN architectures, specifically U-Net, for forest mapping from optical images [39,40]. In [39], the authors used a U-Net to identify instances of a given tree species from WorldView-3 images. Similarly, in [40], the U-Net was trained with the RGB bands and the digital elevation models (DEM) from high resolution UAV imagery. The importance of monitoring urban forests and the lack of studies on using FCNs’ capabilities for this purpose motivated the present study. We propose and evaluate in this paper the use of five state-of-the-art deep learning methods for semantic segmentation of individual tree species identification in an urban context using RGB images derived from UAVs.

Specifically, we focus on identifying the canopy of the threatened species *Dipteryx alata* Vogel, also known as cumbaru. It comes about in midwestern Brazil, and due to its particular shadow and architecture, it is used for afforestation practices over urban areas. This species has a tremendous social and economic relevance for the development of some areas of the Brazilian Cerrado [41]. It has been threatened by extinction according to the IUCN (2020) (The International Union for Conservation of Nature’s Red List of Threatened Species, https://www.iucnredlist.org/species/32984/9741012), which makes its preservation a very important issue since this particular species provides fruits for a large number of bird species.

The main contributions of this work are threefold: (I) to evaluate the capability of deep learning methods to segment individual trees on high spatial resolution RGB/UAV images; (II) to compare five state-of-the-art deep learning semantic segmentation methods, namely U-Net, SegNet, FC-DenseNet, and Deeplabv3+ with the Xception and MobileNetV2 backbone, for the segmentation of cumbaru trees on the aforementioned RGB/UAV imagery; and (III) to assess the improvements of using CRFs as a post-processing step for individual tree level semantic segmentation.

The remainder of this paper is organized as follows: Section 2 describes the study areas and introduces the fundamentals of FCNs, specifically the approaches used in this work. It further presents the protocol followed in our experimental analysis. Section 3 presents and discusses the experimental results. Finally, Section 4 summarizes the main conclusions of this work and points to future directions.

## 2. Materials and Methods

### 2.1. Study Area and Data Acquisition

**Figure 1:**
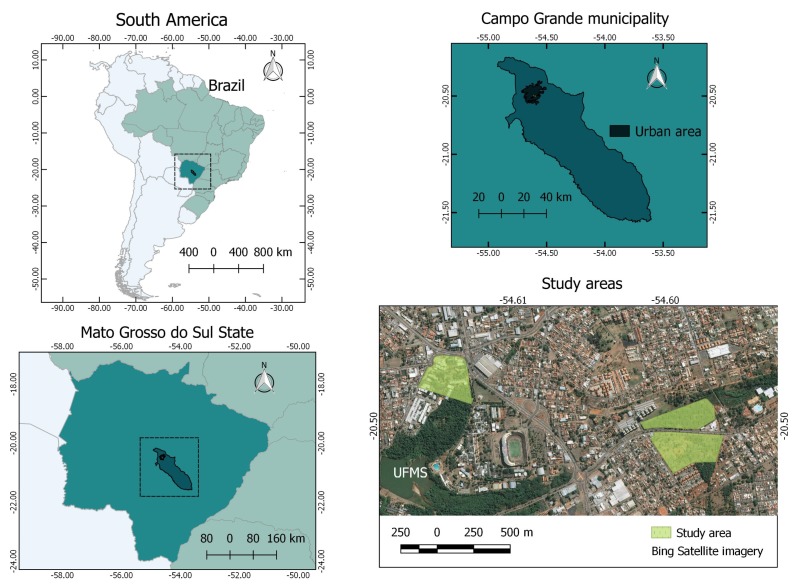
Study area at Campo Grande, Mato Grosso do Sul, Brazil.

The dataset used in this work contained UAV images of Campo Grande municipality, in the state of Mato Grosso do Sul, Brazil (Figure 1). This dataset was a subset of the one presented in [21] and comprised 225 UAV images acquired from 13 August 2018 to 22 September 2018 using a Phantom 4 advanced quadcopter (DJI Innovation Company Inc., Shenzhen, China) in three study areas, depicted in Figure 1. The UAV had an RGB camera with 20 megapixels, a CMOS sensor, a nominal focal length of 8.8 mm, and a field of view of 84°. The dataset is available upon request.

**Figure 2:**
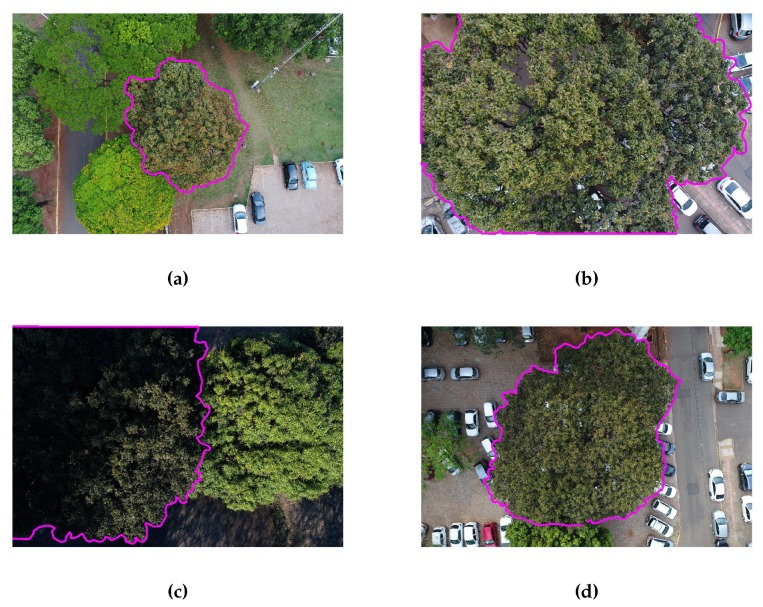
Image samples with the reference tree contour in pink, showing variations in terms of scale (**a**,**b**), illumination (**c**), and different urban patterns (**d**).

The flight height ranged from 20 to 40 m over the targets, which assured a mean ground sample distance (GSD) of approximately 1 cm (Figure 2). An analyst well acquainted with the target area produced the reference masks by delineating manually each single tree. Field inspections were performed to assure data quality.

The images were 5472 × 3648 pixels (20 megapixels) large and represented a wide range of appearances and scale variations. They were acquired at different times of the day and were therefore affected by different illumination conditions. The images were captured over diverse neighborhoods characterized by different urban patterns. The cumbaru class accounted for approximately 44% of the total pixels of the dataset.

### 2.2. Semantic Segmentation Methods

Over the past few years, the fully convolutional networks [33] have gained recognition due to their ability to perform pixel based classification in an end-to-end fashion [42,43]. In this section, we describe succinctly the four FCN architectures assessed in this work for tree segmentation: SegNet, U-Net, FC-DenseNet, and the two variants of the DeepLabv3+ related to the adopted backbone: Xception and MobileNetV2. Finally, we revisit the idea of applying conditional random fields (CRFs) as a post-processing technique to improve the overall segmentation outcome.

#### 2.2.1. U-Net

**Figure 3:**
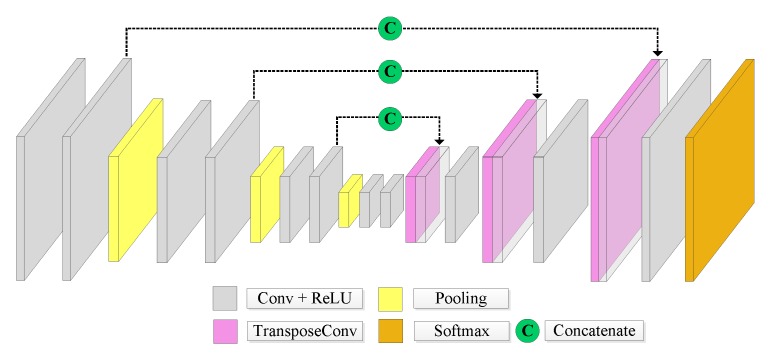
U-Net architecture.

The U-Net (see Figure 3) has an encoder-decoder architecture [34]. The encoder is a stack of convolutional and max-pooling layers. The decoder is a symmetric expanding path that uses learnable deconvolution filters to upsample the feature maps. The main novelty introduced by this network is how the so-called skip connections are used. Specifically, they allow for the concatenation of the output of the transposed convolution layers with the correspondent feature maps of the encoder stage [44]. This step aims at retrieving the fine characteristics learned by the contracting stages to restore the original input image’s spatial resolution [34].

#### 2.2.2. SegNet

**Figure 4:**
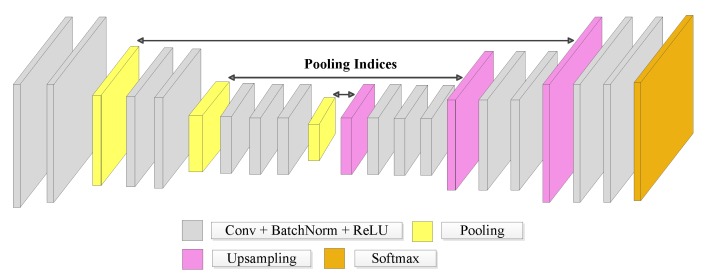
SegNet architecture.

SegNet was also one of the early proposed FCN architectures for semantic segmentation [24]. This network has an encoder and a corresponding decoder path, followed by a final pixel-wise classification layer. The encoder comprises a series of convolutional layers, whose outputs are normalized before being applied to a nonlinear activation function followed by 2 × 2 max-pooling (see Figure 4). A distinguishing characteristic of SegNet is that it keeps the pooling indices, i.e., the position of the cell within each 2 × 2 group of pixels where the max-pooling operation took the maximum from. These indices are forwarded to the correspondent upsampling layer of the decoder stage. Each upsampling step of the decoder stage involves doubling the spatial resolution. The max-pooling indices stored during the encoder phase determine the cell of the corresponding 2 × 2 array at the higher resolution output where each input value is to be loaded. The other three cells of the 2 × 2 array are zeroed. In this way, SegNet seeks to retrieve the input image details lost in the encoder downsampling steps.

#### 2.2.3. FC-DenseNet

**Figure 5:**
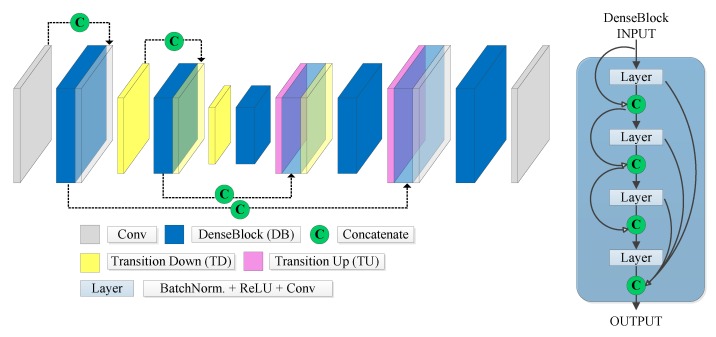
FC-DenseNet architecture.

Based on fully convolutional networks, Jégou et al. [45] extended the DenseNet network [46] by adding an upsampling path to recover the input resolution and proposed the fully convolutional DenseNet (FC-DenseNet). Its architecture is illustrated in Figure 5. The traditional DenseNet is built on the so-called dense blocks. Each dense block layer is composed of batch normalization, followed by a ReLU activation function and a 3 × 3 convolution [45]. The output of a dense block is the concatenation of the outputs of each layer in the current block. Thus, the number of feature maps increases after each layer, by a factor of *k*, a network hyperparameter called growth rate.

FC-DenseNet keeps the dense blocks of [46] and includes the downsampling and upsampling paths with skip connections. The downsampling path consists of dense blocks followed by transition down (downsampling) layers, which are composed of a batch normalization, ReLU activation function, an 1 × 1 convolutional layer, and a 2 × 2 max-pooling operation [45]. Analogously, the decoder consists of dense blocks and transition up (upsampling) layers, which perform a single transposed convolution with stride 2 [47]. Like the U-Net, the skip connections concatenate the feature maps in the upsampling path with the downsampling feature map at the same level. To avoid an excessive growth of feature maps in the upsampling path, the input of the dense block is not concatenated with its output [45].

#### 2.2.4. DeepLabv3+ with the Xception Backbone

Keeping the encoder-decoder structure, the fourth and fifth approaches considered in this work are based on the DeepLabv3+, which represented the state-of-the-art for semantic image segmentation [38] at the time this paper was written. The characteristic of this network is the atrous convolution, also known as dilated convolution, which operates on an input feature map (x) as follows:

(1) $$y\left(i\right)=\sum_{k}x\left[i+r*k\right]w\left[k\right]$$

where *i* is the location in the output feature map y, w is a convolution filter, and *r* is the atrous rate that determines the stride in which the input signal is sampled [48].

Another characteristic of this method is the atrous spatial pyramid pooling (ASPP). This technique was introduced in [38] and involves employing atrous convolution in parallel as a strategy to extract features at multiple scales and to alleviate the loss of the spatial information due to prior pooling or convolutions with striding operations [48]. In relation to conventional architectures, ASPP allows increasing the field of view and thus the spatial context considered at each layer with a smaller increase in the number of parameters and in computational complexity [38].

It is worth mentioning that DeepLabv3+ inherited the separable convolutions introduced in its predecessor, the DeepLabv3 version. While standard convolution performs the channel-wise and spatial-wise computation in one step, depthwise separable convolution splits the computation into two steps: depthwise convolution and pointwise convolution. Depthwise convolution performs an independent spatial convolution per each input channel using only a convolutional filter, while pointwise convolution is used to combine the output of the depthwise convolutions [49]. In comparison with the standard convolution, these operations reduce the number of parameters and the computation cost, maintaining similar accuracies.

**Figure 6:**
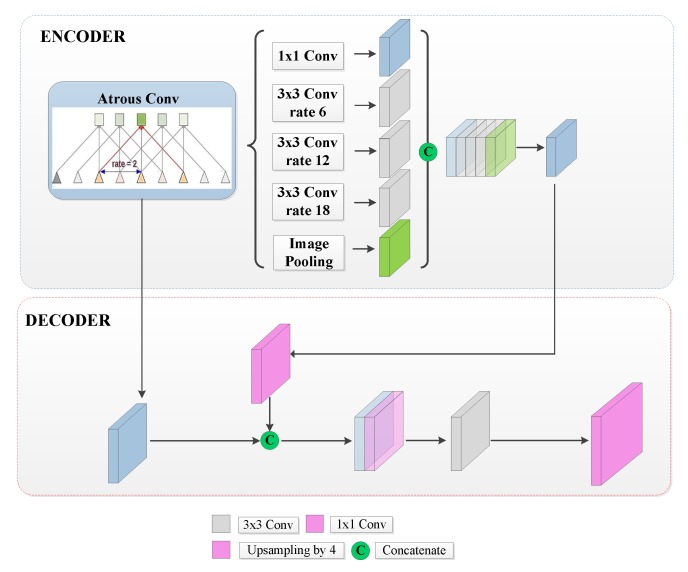
DeepLabv3+ architecture.

Furthermore, the DeepLabv3+ version modifies the Xception model presented in [50], replacing all max-pooling operations by depthwise separable convolutions [48]. In the decoder stage, the features obtained from the encoder are upsampled by a factor of 4 and then concatenated with the corresponding low level features [51]. In order to make better use of higher semantic features extracted by the encoder, an 1 × 1 convolution is employed to reduce the number of channels. After the concatenation, a 3 × 3 convolution is applied to refine the features, ending with another bilinear upsampling by a factor of 4 to obtain the resolution of the input image [48,52]; see Figure 6.

#### 2.2.5. DeepLabv3+ with the MobileNetV2 Backbone

We also evaluated a variant of the DeepLabv3+ using a MobileNetV2 backbone [53]. This model was proposed to reduce computational complexity so that DeepLabv3+ could run on mobile devices. The key concept behind MobileNetV2 is the introduction of the inverted residual blocks in the bottleneck of the main architecture.

In conventional residual blocks, the depth of the tensor comprising the input feature maps is first reduced by a 1 × 1 convolution whose output feeds a subsequent 3 × 3 convolution. Prior to adding the result to the input feature map, another 1 × 1 convolution is carried out to match the depth of input feature maps. The inverted residual block presented in [53] works the other way around. It first applies a 1 × 1 convolution to increase the depth of feature maps’ tensor, followed by a 3 × 3 depthwise convolution. A subsequent 1 × 1 convolution compresses the resulting tensor back to the depth of the input feature map. This scheme involves considerably fewer parameters than the conventional residual block and is more efficient computationally.

### 2.3. Conditional Random Field

To improve semantic segmentation and labeling accuracy, probabilistic graphical models have been used as post-processing. Markov random fields (MRFs) and particularly conditional random fields (CRFs) have achieved widespread success in this task [54,55,56].

While deep neural networks have proven effective in learning features from a small field of view, they fail to capture global context information. To address this issue, approaches have been proposed to combine the effectiveness of CNNs to learn discriminatory characteristics, with the CRF’s ability to model broad spatial contexts. CRF approaches semantic labeling as a probabilistic inference problem assuming that neighboring pixels tend to share the same class label unless their descriptors differ significantly.

Given the set of pixels i∈S of an image, let $x={\left\{x_{i}\right\}}_{i\in S}$ be the observed data and $y={\left\{y_{i}\right\}}_{i\in S}$ its corresponding labels, where $y_{i}$ may take values in $\left\{l_{1},\ldots ,l_{m}\right\}$ and *m* is the number of available classes. A CRF models the posterior probability P(y|x) of the set of labels y given the image data x as follows:

(2) $$P\left(y|x\right)\propto \exp\left\{-\left[\sum_{i\in S}A\left(y_{i},x\right)+\sum_{i\in S}\sum_{j\in N_{i}}I\left(y_{i},y_{j},x\right)\right]\right\},$$

where $A(y_{i},x)$ and $I(y_{i},y_{j},x)$ stand for the association and iteration potentials, also named unary and pair-wise terms, respectively. The optimum class assignment $\hat{y}$ given x is the one that maximizes the posterior, i.e.,

(3) $$\hat{y}=\arg\min_{y}\left(\sum_{i\in S}A\left(y_{i},x\right)+\sum_{i\in S}\sum_{j\in N_{i}}I\left(y_{i},y_{j},x\right)\right).$$

The unary term relates to the posterior probability that a pixel *i* takes a label $y_{i}$ given the data x. In this work, the posteriors are given by one of the FCNs described in the foregoing sections. Consequently, the unary of any pixel will consider a limited spatial context determined by the FCN largest receptive field. On the other hand, the pair-wise term expresses how labels at neighboring pixels, *i* and $j\in N_{i}$, interact given the observed data x, where $N_{i}$ is the neighborhood of pixel *i*. Notice that the pair-wise term allows for non-neighboring pixels to interact through a sequence of intermediate neighboring pixels. In this way, the CRF model is able to capture information of a context as large as the image itself.

Actually, using CRF inference as post-processing does not exploit the full potential of CRF. This is mainly because CNN is trained with no regard to the CRF post-processing. Nevertheless, it has been shown to be beneficial when combined with some of the aforementioned FCN architectures. However, this accuracy gain comes at the cost of increased computational complexity both for training and inference.

### 2.4. Experimental Evaluation

We started our experiments with the networks’ configurations exactly as defined in the corresponding original papers. Next, we varied some of their hyper-parameters, such as the number of layers, operations per layer, and the number and size of kernels, aiming to fine tune each network to the target application. After these preliminary experiments, we selected for SegNet, U-Net, and FC-DenseNet the architectures described in Table 1. For both DeepLabv3+ variants, we adopted the original design as proposed in [48,53]. Table 2 shows for each network the total number of parameters that must be estimated by supervised training.

As for the post-processing, we adopted a fully connected CRF. Since in this case, all pixels were connected to all other pixels in the image, Equation (3) took the form:

(4) $$\hat{y}=\arg\min_{y}\left(\sum_{i\in S}A\left(y_{i},x\right)+\sum_{i,j\in S}I\left(y_{i},y_{j},x\right)\right)$$

We defined as association potential $A\left(y_{i},x\right)=-\log P\left(y_{i}|x_{i}\right)$, where $P(y_{i}|x_{i})$ denotes the posterior given the FCNs tested in this work. As in [25] and [56], we used for the pair-wise term the following expression:

(5) $$\begin{array}{c} I\left(y_{i},y_{j},x\right)=\mu \left(y_{i},y_{j}\right)[w_{1}\exp\left(-\frac{\left|\right|c_{i}-c_{j}{\left|\right|}^{2}}{2{\sigma_{\alpha }}^{2}}-\frac{\left|\right|x_{i}-x_{j}{\left|\right|}^{2}}{2{\sigma_{\beta }}^{2}}\right)\\ +w_{2}\exp\left(-\frac{\left|\right|c_{i}-c_{j}{\left|\right|}^{2}}{2{\sigma_{\gamma }}^{2}}\right)] \end{array}$$

where $\mu (y_{i},y_{j})=1$ if $y_{i}\neq y_{j}$, and zero otherwise, $x_{i,j}$ represents the observed data at pixel i,j, and $c_{i,j}$ denotes the pixel spatial coordinates. The hyperparameters $w_{1}$, $w_{2}$, $\sigma_{\alpha }$, $\sigma_{\beta }$ and $\sigma_{\gamma }$ were set to 1, 1, 80, 13, and 10, respectively, which corresponded to their default values as proposed in [56]. For our experiments, we adapted the fully CRF code available at https://github.com/Golbstein/Keras-segmentation-deeplab-v3.1/blob/master/utils.py.

The networks were implemented using the Keras deep learning framework [57] on a system with the following configuration: Intel(R) Core(TM) i7 processor, 64 GB of RAM, and NVIDIA GeForce GTX 1080Ti GPU. Due to the limitations of the GPU memory, images were divided into patches of 512×512 pixels for all models. The final segmentation of the entire image was the mosaic of all patch-wise segmentation outcomes. Before starting the experiments, the images were first normalized in the range [0-1] for the U-Net, SegNet, and FC-DenseNet. For the DeepLabv3+ variants, no normalization was applied.

To evaluate the generalization of the models, we applied a fivefold cross-validation for each method. Thus, for each fold, the dataset was randomly split into three disjoint sets: 70% for training, 10% for validation, and 20% for testing.

All models were trained from scratch for up to 50 epochs using the Adam optimizer with a learning rate of 0.0001. The decay of the first and second moments were set as described in the original paper [58]. Early stopping was used to avoid overfitting. Training stopped when the performance in the validation set degraded over ten consecutive epochs. In the end, the model that exhibited the best performance in the validation set across all executed epochs was kept for the test phase.

We empirically adjusted the batch size for each model individually, also taking into account the GPU memory demand and availability. The batch size was set to 16 for the U-Net, 8 for the SegNet, 6 for the FC-DenseNet, 2 for the DeepLabv3+ with the Xception backbone, and 6 for the DeepLabv3+ with MobileNetV2. We tested Deeplabv3+ with an output stride equal to 16 with the atrous rate of (6,12,18), similarly as in [38].

Referred to the growth rate parameter of the FC-DenseNet, it was empirically set to 8.

### 2.5. Evaluation Metrics

Accuracy is reported henceforth in terms of three metrics: overall accuracy (OA), F1-score (F1), and intersection over union (IoU).

The overall accuracy is given by:

(6) $$OA=\frac{TP+TN}{TP+TN+FP+FN}$$

where TP,TN,FP,FN stand for the number of true positives, true negatives, false positives and false negatives, respectively. In our analysis, positives and negatives refer to the pixels assigned by the underlying classifier to the cumbaru and non-cumbaru classes, respectively. Such positives and negatives are true or false, depending on whether or not they agree with the ground truth, respectively.

The F1-score is defined as:

(7) $$F1=2\times \frac{P\times R}{P+R},$$

where *P* and *R* stand for precision and recall, respectively, and are given by the ratios [59]:

(8) $$P=\frac{TP}{TP+FP}$$

(9) $$R=\frac{TP}{TP+FN}$$

For semantic segmentation tasks, the intersection over union (IoU), also known as the Jaccard index, has often been used as an accuracy metric. IoU is given by the ratio of the number of pixels present both in the reference and in the prediction masks to the total number of pixels present across both masks [60], formally:

(10) $$IoU=\frac{\left|Reference\cap Prediction\right|}{\left|Reference\cup Prediction\right|}$$

## 3. Results and Discussion

In this section, we present the results of the experimental evaluation of the selected semantic segmentation approaches in terms of OA, F1, and IoU (Section 3.1), as well as a visual analysis of the segmentation outcomes (Section 3.2). Finally, we assess the computational efficiency of each method in Section 3.3.

### 3.1. Performance Evaluation

**Figure 7:**
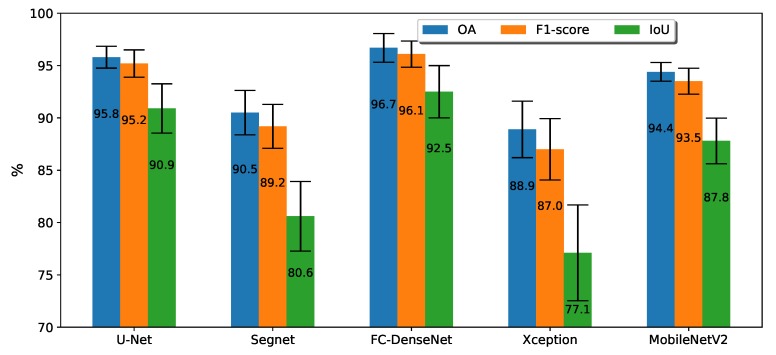
Mean of the overall accuracy (OA), F1, and IoU in the fivefold cross-validation for the all FCN architectures.

Figure 7 shows the average results over fivefold cross-validation for each method. Semantic segmentation methods performed well in the task, achieving OA and an F1-score above 85% and IoU above 75%. The plot also shows pictorially the standard deviation for each metric across the folds. On the whole, the standard deviation was about ±1% for OA and a little larger for F1 and IoU.

FC-DenseNet was the most accurate among the tested architectures. It reached on average OA = 96.7%, F1 = 96.1%, and IoU = 92.5%. It outperformed the second ranked network, the U-Net, in 0.9% in terms of OA and F1 respectively, and 1.6% for IoU. Shortly behind U-Net came DeepLabv3+ in the MobileNetV2 version. The differences between these two architectures in all three metrics were about 1.4%, 1.7%, and 3.1% in terms of OA, F1, and IoU, respectively. Figure 7 also shows the standard deviation around the mean values recorded by each architecture along the five folds for all three metrics. These three high ranked methods also presented lower dispersion than the other two in our experiments, behaving fairly stably across the folds. The low variation across the folds observed in our experiments is an indication of the better generalization ability of these three methods.

SegNet’s architecture ranked forth, staying 3.9%, 4.3%, and 7.2% behind DeepLabv3+ MobileNetV2, in terms of OA, F1, and IoU, respectively. The range within which performance varied in our experiments confirmed that SegNet stood behind the first three architectures in the ranking. The inferior SegNet’s results were due to the way it recovered the original image resolution in the network expansion stage. SegNet employed interpolation, while U-Net and FC-DenseNet used transposed convolution. Moreover, the skip connections of U-Net and FC-DenseNet were more effective in recovering high resolution spatial details than the consideration of pool indices by SegNet.

In the recent few years, the DeepLabv3+ Xception has been regarded as the state-of-the art in semantic segmentation. Nevertheless, it achieved in our experiments the worst performance among all tested architectures, both in terms of absolute average accuracies and in terms of variability across the five folds. The MobileNetV2 variant overcame the Xception counterpart by approximately 5.5% for OA, 6.5% for F1-score, and 10.7% for IoU. According to Table 2, DeepLabv3+ Xception comprised about 20 times more parameters than MobileNetV2. This implies a much higher demand for training samples that may not have been met by our dataset, causing DeepLabv3 + Xception to perform below its potential.

**Figure 8:**
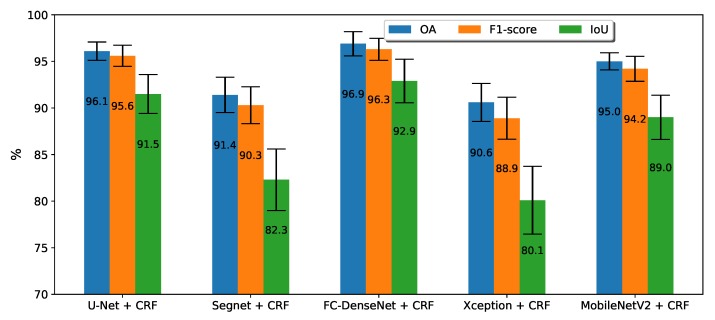
Average of the accuracy, F1-score, and IoU in fivefold cross-validation for all the methods using CRF.

Figure 8 shows the performance after post-processing the results produced by each network with a fully connected CRF. Compared with the results of Figure 7, CRF brought just a slight improvement of the metrics for all methods. The profile in Figure 8 is quite similar to that of Figure 7. Again, after CRF post-processing, FC-DenseNet was the best performing architecture, followed by U-Net, DeepLabv3+ MobileNetV2, and then SegNet with DeepLav3+ Xception as the worst performing network.

**Figure 9:**
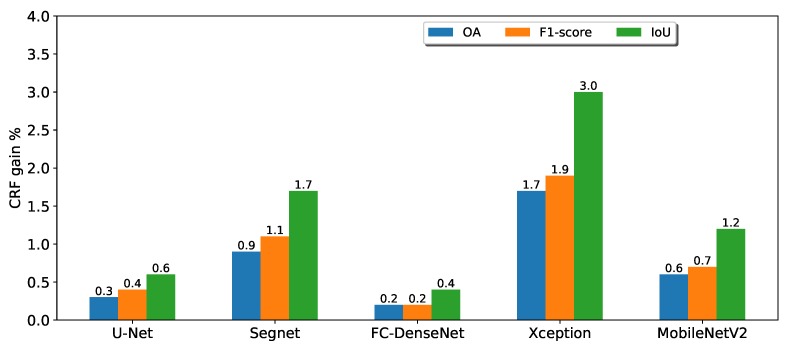
Performance gains due to CRF in terms of overall accuracy, F1-score, and IoU.

In order to better visualize the benefits of post-processing, Figure 9 shows just the accuracy gain brought by CRF. DeepLav3+ with the Xception backbone was the network that most profited from CRF. In second and third place were SegNet and DeepLabv3+ MobileNetV2, respectively. The gains for U-Net and FC-DenseNet were considerably lower, below 0.6%.

The results of the confusion matrix for all the FCN methods with and without CRF are presented in Appendix A. The true positives, true negatives, false positives, and false negatives values represent the mean value among the five folds.

### 3.2. Visual Analysis

**Figure 10:**
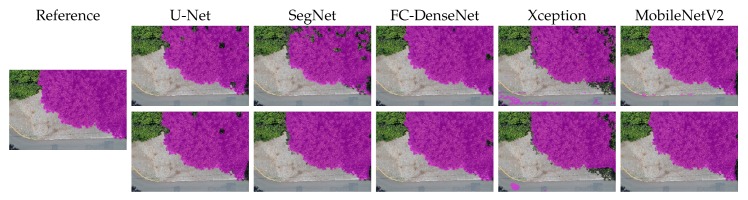
Sample Segmentation 1 prior (first row) and after CRF post-processing (second row).

**Figure 11:**
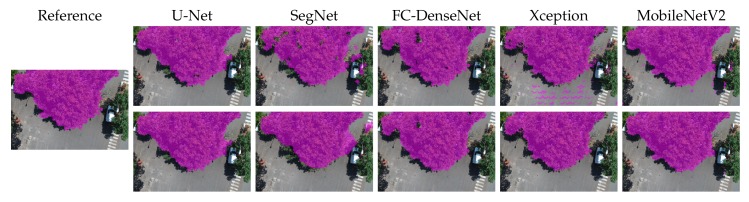
Sample Segmentation 2 prior (first row) and after CRF post-processing (second row).

**Figure 12:**
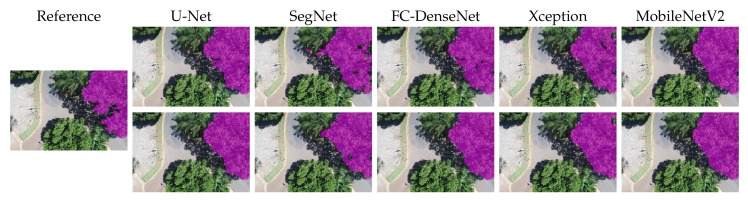
Sample Segmentation 3 prior (first row) and after CRF post-processing (second row).

**Figure 13:**
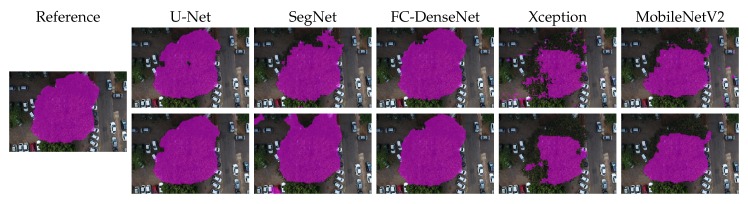
Sample Segmentation 4 prior (first row) and after CRF post-processing (second row).

**Figure 14:**
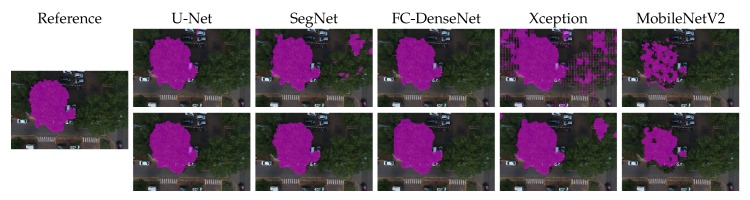
Sample Segmentation 5 prior (first row) and after CRF post-processing (second row).

Figure 10, Figure 11, Figure 12, Figure 13 and Figure 14 show the outcomes of all methods for five sample images of our dataset. References are shown on the left as the ground truth mask overlaid on the input image. The next four columns on the right contain the segmentation produced by each network, whereby the upper and lower rows correspond to the outcome prior to and after CRF post-processing, respectively.

We first analyzed the results prior to CRF post-processing. More than the U-Net architecture, SegNet tended to produce holes in the canopy region. This effect is especially apparent in Figure 10, Figure 11, Figure 12 and Figure 13. Figure 11 and Figure 14 show that SegNet also produced a number of false positives in some images. Often, it also failed to detect the canopy edges, as exemplified in Figure 13.

The visual inspection shows that U-Net outperformed SegNet in virtually all test images of the database. Actually, the MobileNetV2 version of DeepLabv3+ generated fewer holes than U-net in some images. However, DeepLabv3+ MobileNetV2 was more prone to produce false positives (see Figure 10, Figure 11, and Figure 13).

Both DeepLabv3+ variants often failed over dark canopy regions as exemplified by Figure 13 and Figure 14. These same figures show that SegNet performed better than the DeepLabv3+ variants over dark areas, but not as well as the other networks. FC-DenseNet and U-Net’s results were freer from false positives and false negatives in dark canopy regions.

The figures also reveal that the Xception variant of DeepLabv3+ achieved the poorest performance among the evaluated architectures, due to holes (Figure 10 and Figure 12), false positives (Figure 10, Figure 11, and Figure 13), and poorly classified dark areas (Figure 13 and Figure 14). As stated before, we argue that the MobileNetV2 variant delivered better results than the Xception counterpart thanks to its lower complexity. This is attested to by all the visual results presented in this section.

The effects of CRF post-processing on the segmentation outcome can be observed by comparing the results shown in the upper and lower rows of Figure 10 to Figure 14. CRF acted by smoothing the class labels produced by the networks. It was particularly effective at filling holes and suppressing regions of false positives. That was related to the fact that both DeepLabv3+ variants profited more than all other networks from CRF post-processing as indicated in Figure 9. However, these errors corrected by CRF came about as few small regions that represented in terms of the number of pixels a small proportion of the entire image. Thus, although CRF contributed to producing cleaner segmentation outcomes, such improvements did not manifest in a significant change in the accuracy metrics adopted in this evaluation as shown in Figure 9. This could be observed even in the results of FC-DenseNet, the best performing network among all tested approaches. Note in Figure 10 to Figure 13 that CRF managed to remove small holes left by FC-DenseNet in the canopy region.

### 3.3. Computational Complexity

In this section, we compare the methods in terms of computational efficiency and computational load for training and inference. Table 3 presents the average training and inference times measured on the hardware infrastructure described in Section 2.4. The training time represents the mean time among the five folds for each method. The mean inference time stands for the average time taken by each model to make predictions image-by-image in the test set for one fold.

Considering that the methods were trained with the same optimizer and learning rates, these results were highly correlated with the network depth and the selected batch size. For instance, the DeepLabv3+ Xception network was much deeper than the others. As a consequence, it took longer than the other networks for training and inference. DeepLabv3+ MobileNetV2 was also deeper than the other three methods, which implied longer inference times, which may be critical for real-time applications. On the other hand, this network was one of the fastest in the training phase because, despite its depth, it had a small number of parameters. SegNet was relatively fast for both training and inference. However, its results in terms of the evaluated metrics were comparatively low, as mentioned in Section 3.1. Finally, the CRF post-processing implied very long additional execution times, which may prevent its usage in applications where the processing time is crucial.

## 4. Conclusions and Research Perspective

In this work, we proposed and evaluated the use of state-of-the-art fully convolutional networks for semantic segmentation of a threatened tree species using high spatial resolution RGB images acquired by UAV platforms. Five architectures were tested: SegNet, U-Net, FC-DenseNet, and two DeepLabv3+ variants, specifically Xception and MobileNetV2. The analysis was conducted on a dataset that represented an urban context. The experiments demonstrated that networks could learn the distinguishing features of the target tree species in a supervised way. This fact indicated that the tested FCN designs could delineate other tree species, provided that enough representative labeled samples are available for training.

Among the tested networks, FC-DenseNet attained the best performance achieving 96.7%, 96.1%, and 92.5% in terms of overall accuracy, F1-score, and IoU. Ranked second and third were U-Net and DeepLabv3+ MobileNetV2, respectively, with a difference of 1.4%, 1.7%, and 3.1% for overall accuracy, F1-score, and IoU, followed by the SegNet. The lowest accuracy scores were achieved by DeepLabv3+ Xception with 88.9% for overall accuracy, 87.1% for the F1-score, and 77.1% for IoU. Notably, this was the most complex of all evaluated networks. It contained about 100 times more learnable parameters than FC-DenseNet, the best performing network.

As for the computational efficiency, FC-DenseNet and DeepLabv3+ Xception were again the best and the worst performing networks, respectively, in terms of inference times.

We also observed in our study that post-processing the networks’ outcomes by a fully connected CRF was beneficial in nearly all cases. However, the impact on overall accuracy metrics was often numerically modest, because CRF generally fixed errors in small image regions. Yet, the improvement in segmentation quality was usually significant, as evidenced by visual inspection. The price for such accuracy gain was the comparatively long CRF processing time, about 30 times the FC-DenseNet’s inference time.

DeepLabv3 + Xception was by far the most complex among the networks to be tested. Though it was regarded in the literature as staying amongst the top performing FCNs, DeepLabv3+ Xception achieved the worst accuracy compared to all tested networks. This finding suggested that the training data fell short to estimate DeepLabv3+ Xception’s parameters properly. Even the simpler MobileNetV2 version, which involved just 1/20 of learnable parameters, surpassed the Xception version in all experiments.

We also noticed that DeepLabv3+ Xception benefited from CRF more than all other architectures. In the continuation of this research, we intend to verify if CRF is generally able to mitigate the problem of scarce training data for FCN based semantic segmentation. Additionally, we aim to investigate the application of morphological operations as a post-processing alternative. Another issue that deserves further analysis concerns the generalizability of these methods. Unfortunately, the number and diversity of annotated databases available for this purpose are still limited. We are currently working on building a more diverse database in terms of sensors, tree species, and climate characteristics.

## Figures and Tables

**Table 1:** Details of the SegNet, U-Net, and FC-DenseNet architectures used in the experimental analysis.

	SegNet		U-Net		FC-DenseNet	
	Layer	Kernel No.	Layer	Kernel No.	Layer	Kernel No.
Encoder	2 × SB + pool	32	UB + pool	32	conv1	32
	2 × SB + pool	64	UB + pool	64		
	3 × SB + pool	128	4 × (UB + pool)	128	8 × (DB + TD)	48
	3 × SB + pool	256	UB + pool	256		
	3 × SB + pool	512	UB	512	DB	176
Decoder	Up + 2 × SB	512	TC + Concat.+ UB	256		
	SB + Up	256	TC + Concat. + UB	128		
	2 × SB	256	TC + Concat. + UB	128		
	SB + Up	128	TC + Concat. + UB	128	8 × (TC + DB)	192
	2 × SB	128	TC + Concat. + UB	128		
	SB + Up	64	TC + Concat. + UB	64		
	SB	64	TC + Concat. + UB	32		
	SB + Up	32				
	SB	32				
	conv2 (1 × 1)	2	conv2 (1 × 1)	2	conv2 (1 × 1)	2
	Softmax		Softmax		Softmax	

For SegNet, we adopted the following notations: SB stands for the SegNet block (3 × 3 convolution + batch normalization + ReLU) and Up for unpooling layers. Concerning the U-Net, UB stands for U-Net block (2 × (3 × 3 convolution + ReLU)), and TC denotes a 3 × 3 transposed convolution. The FC-DenseNet was built from dense blocks (DB) of 2 layers, where each layer stands for (ReLU + 3 × 3 convolution). The transition down (TD) operation represents (ReLU + 1 × 1 convolution).

**Table 2:** Number of parameters of each network.

Method	Parameters
U-Net	11M
SegNet	16M
FC-DenseNet	0.4M
DeepLabv3+ (Xception)	41M
DeepLabv3+ (MobileNetV2)	2M

**Table 3:** Average processing time for each method.

Method	Training	Inference
	Time (h:min)	Time (s)
U-Net	13:15	1.15
SegNet	09:12	1.17
FC-DenseNet	15:03	1.14
DeepLabv3+ (Xception)	20:33	4.44
DeepLabv3+ (MobileNetV2)	10:46	2.26
CRF	-	35.17

## Appendix A

**Table A1:** Confusion matrix for the U-Net without CRF (left) and with CRF (right). The values are normalized by the total of pixels.

		True				True	
		Cumbaru	Background			Cumbaru	Background
		(+)	(−)			(+)	(−)
**Predicted**	Cumbaru (+)	0.42	0.02	**Predicted**	Cumbaru (+)	0.43	0.02
	Background (−)	0.02	0.54		Background (−)	0.02	0.53

**Table A2:** Confusion matrix for the SegNet without CRF (left) and with CRF (right). The values are normalized by the total of pixels.

		True				True	
		Cumbaru	Background			Cumbaru	Background
		(+)	(−)			(+)	(−)
**Predicted**	Cumbaru (+)	0.39	0.05	**Predicted**	Cumbaru (+)	0.4	0.04
	Background (−)	0.05	0.51		Background (−)	0.04	0.52

**Table A3:** Confusion matrix for the FC-DenseNet without CRF (left) and with CRF (right). The values are normalized by the total of pixels.

		True				True	
		Cumbaru	Background			Cumbaru	Background
		(+)	(−)			(+)	(−)
**Predicted**	Cumbaru (+)	0.41	0.01	**Predicted**	Cumbaru (+)	0.41	0.01
	Background (−)	0.02	0.56		Background (−)	0.02	0.56

**Table A4:** Confusion matrix for the DeepLabv3+ Xception version without CRF (left) and with CRF (right). The values are normalized by the total of pixels.

		True				True	
		Cumbaru	Background			Cumbaru	Background
		(+)	(−)			(+)	(−)
**Predicted**	Cumbaru (+)	0.37	0.02	**Predicted**	Cumbaru (+)	0.38	0.01
	Background (−)	0.09	0.52		Background (−)	0.08	0.53

**Table A5:** Confusion matrix for the DeepLabv3+ MobileNetV2 version without CRF (left) and with CRF (right). The values are normalized by the total of pixels.

		True				True	
		Cumbaru	Background			Cumbaru	Background
		(+)	(−)			(+)	(−)
**Predicted**	Cumbaru (+)	0.41	0.02	**Predicted**	Cumbaru (+)	0.41	0.02
	Background (−)	0.03	0.54		Background (−)	0.03	0.54
